# Effect of Essential Oils and Vacuum Packaging on Spoilage-Causing Microorganisms of Marinated Camel Meat during Storage

**DOI:** 10.3390/foods10122980

**Published:** 2021-12-03

**Authors:** Tareq M. Osaili, Fayeza Hasan, Anas A. Al-Nabulsi, Dinesh Kumar Dhanasekaran, Reyad Shaker Obaid, Mona S. Hashim, Hadia M. Radwan, Leila Cheikh Ismail, Haydar Hasan, Moez Al-Islam E. Faris, Farah Naja, Ioannis N. Savvaidis, Amin N. Olaimat, Mutamed Ayyash, Richard Holley

**Affiliations:** 1 Department of Clinical Nutrition and Dietetics, College of Health Sciences, University of Sharjah, Sharjah P.O. Box 27272, United Arab Emirates; robaid@sharjah.ac.ae (R.S.O.); mhashim@sharjah.ac.ae (M.S.H.); hradwan@sharjah.ac.ae (H.M.R.); lcheikhismail@sharjah.ac.ae (L.C.I.); haidarah@sharjah.ac.ae (H.H.); mfaris@sharjah.ac.ae (M.A.-I.E.F.); fnaja@sharjah.ac.ae (F.N.); 2 Sharjah Institute for Medical Research, University of Sharjah, Sharjah P.O. Box 27272, United Arab Emirates; U00040620@sharjah.ac.ae (F.H.); ddhanasekaran@sharjah.ac.ae (D.K.D.); 3 Department of Nutrition and Food Technology, Faculty of Agriculture, Jordan University of Science and Technology, Irbid 22110, Jordan; anas_nabulsi@just.edu.jo; 4 Department of Environmental Health Sciences, College of Health Sciences, University of Sharjah, Sharjah P.O. Box 27272, United Arab Emirates; isavvaidis@sharjah.ac.ae; 5 Department of Chemistry, School of Natural Sciences, University of Ioannina, 45110 Ioannina, Greece; 6 Department of Clinical Nutrition and Dietetics, Faculty of Applied Medical Sciences, The Hashemite University, Zarqa 13133, Jordan; aminolaimat@hu.edu.jo; 7 Department of Food Science, College of Agriculture and Veterinary Medicine, United Arab Emirates University (UAEU), Al Ain P.O. Box 15551, United Arab Emirates; mutamed.ayyash@uaeu.ac.ae; 8 Department of Food and Human Nutritional Sciences, University of Manitoba, Winnipeg, MB R3T 2N2, Canada; rick_holley@umanitoba.ca

**Keywords:** antimicrobial, red meat, temperature abuse, refrigeration, marination, vacuum packaging

## Abstract

The use of essential oils (EOs) and/or vacuum packaging (VP) with meats could increase product shelf-life. However, no studies investigating the effect of EOs and VP on camel meat background microbiota have been conducted previously. The study aimed to analyze the antimicrobial effect of essential oils (EOs) carvacrol (CA), cinnamaldehyde (CI), and thymol (TH) at 1 or 2% plus vacuum packaging (VP) on the growth of spoilage-causing microorganisms in marinated camel meat chunks during storage at 4 and 10 °C. VP is an effective means to control spoilage in unmarinated camel meat (CM) and marinated camel meat (MCM) compared to aerobic packaging (AP). However, after EO addition to MCM, maximum decreases in spoilage-causing microorganisms were observed under AP on day 7. Increasing the temperature from 4 to 10 °C under AP increased the rate of spoilage-causing bacterial growth in CM and MCM; however, EOs were more effective at 10 °C. At 10 °C the maximum reductions in total mesophilic plate counts, yeast and molds, mesophilic lactic Acid bacteria, *Enterobacteriaceae*, and *Pseudomonas* spp. were 1.2, 1.4, 2.1, 3.1, and 4.8 log CFU/g, respectively. Incorporating EOs at 2% in MCM, held aerobically under temperature abuse conditions, delayed spoilage.

## 1. Introduction

The world production of meat is estimated to be around 340 million tonnes per year. Average annual consumption is estimated at 43 kg meat/person, with the level being even higher in developed countries [1]. Camelids are traditionally reared in the Middle East for meat and milk due to their ability to survive harsh desert conditions [2]. The demand for camel meat is increasing rapidly, as evidenced by the fact that camel meat production around the world in the year 2000 was 329,151 tonnes, but by 2019 it was projected to reach 653,003 tonnes [3]. The rise in demand is probably associated with the perception that camel meat is healthier compared to others in terms of fat, vitamin, mineral and amino acid content [4].

Although meat plays a pivotal role in the diet mainly due to its protein content, it is prone to spoil if not treated properly. The United States Department of Agriculture reported that as much as 52.6 million tonnes of meat produced worldwide is wasted [5]. Although there are many physical and chemical factors that can contribute to spoilage, microbial spoilage is the most hazardous [6]. The high water activity (a_w_), apt pH, and protein and mineral content found in meat constitute an excellent medium for microorganisms to proliferate [6]. If microorganisms such as lactic acid bacteria (LAB), *Enterobacteriaceae* (EN), *Pseudomonas* (PS), and yeast and molds (Y&M) grow, they can result in early spoilage [7].

There are various methods by which spoilage-causing microorganisms can be controlled. Two such techniques that have gained a lot of attention, probably due to their ease of use and efficacy, are essential oils (EOs) and vacuum packaging (VP). EOs such as carvacrol (CA) and thymol (TH) have been observed to exert their antimicrobial effect by disrupting/permeating the cell membrane structure of microorganisms [8]. Vacuum packaging creates an anaerobic environment around contaminants, preventing the growth of strictly aerobic microorganisms and delaying the growth of those that are facultative [9]. Many studies have discussed their interactive inhibitory action against spoilage-causing microbiota [10,11]. A shelf-life increase of up to 6 d has been reported in chicken marinated with oregano oil stored at 4 °C under vacuum [12]. A meta-analysis revealed that selected EOs (origanum, zingiberaceae, and thymus), when correctly applied, reduced spoilage in chilled, stored seafood by 2.5 to 5 times compared to normal refrigerated conditions [13].

A recent trend observed in many food markets is the availability of ready-to-eat, marinated meat products. Although the majority are usually frozen, meat marinated with recipes that include ingredients such as yoghurt cannot be frozen. This is because water will separate from the yoghurt upon thawing and create an unappealing product. In regional markets in Gulf Cooperation Council (GCC) countries, such products are usually stored aerobically with refrigeration, and fresh batches need to be marinated daily or every two/three days. The use of EOs and/or VP with these meats could increase product shelf-life. In addition, it would eliminate the requirement for the daily marination of fresh meat and reduce waste should the product not sell within a day or two of its preparation. Moreover, the VP of meat can also reduce transportation inputs, permit more effective marketing, as well as increase product shelf-life [14]. To the best of our knowledge, no such study investigating the effect of EOs on camel meat background microbiota has been conducted previously.

Hence, the aim of this study was to evaluate the behavior of spoilage-causing microorganisms during storage in marinated camel meat treated with carvacrol (CA), cinnamaldehyde (CI), and thymol (TH) and held under aerobic or VP conditions. Yoghurt-marinated meat was stored at 4 °C to mimic conditions commonly found in the marketplace. The meat was also stored at 10 °C so as to better estimate the effect of EOs and/or VP on microbial growth in cases of mild temperature abuse.

## 2. Materials and Methods

### 2.1. Camel Meat Samples

On the same day of experiments, fresh camel leg meat was obtained from a local butcher shop. Multiple samples were taken on different days. The meat was transported to the Nutrition and Food Research laboratory, University of Sharjah in an icebox to maintain refrigeration. Using a knife sanitized with 70% (*v*/*v*) alcohol and wiped with disposable tissue, the camel meat was portioned into cubes weighing 10.0 ± 0.1 g. Then the meat cubes were stored at 4 °C in a polystyrene tray covered with plastic cling wrap.

### 2.2. Marinade Preparation

A marinade was prepared as per a previously published protocol [15]. It consisted of (g/100 g meat): 20.0 full-fat yogurt, 4.0 crushed tomato, 14.0 crushed onion, 4.0 olive oil, 4.0 vinegar, 1.9 salt, 1.6 kiwi fruit, 1.5 paprika powder, and 0.7 black pepper. For every 100 g of meat, 52 g marinade was used. The fruit and vegetables were washed, and all marinade ingredients were aseptically weighed and mixed in a sterilized stainless-steel bowl.

### 2.3. Essential Oil (EO) Preparation

Active EO components were purchased from Sigma-Aldrich, Germany (Carvacrol, CA ≥ 99%, CAS Number 499-75-2), including thymol (TH ≥ 99%, CAS Number 89-83-8) and trans-cinnamaldehyde (CI ≥ 95%, CAS Number 14371-10-9). Preparation was performed at a final concentration of 1% and 2% [15]. These concentrations have been associated with a reduction in microbial numbers in previous studies [15,16].

### 2.4. Preparation of Treatment and Packaging of the Samples

The camel meat samples were randomly divided into eight individual treatments. All analyses were conducted in triplicate. Meat samples without marination (CM) and marinated camel meat (MCM) were considered as control 1 and 2, respectively. To the MCM, 1 or 2% each of CA, CI, or TH was individually added. Care was taken to mix the meat thoroughly. All work was performed under sterile conditions in a biosafety cabinet (5’ Purifier logic+ class II, Labconco, MO, USA). The treated products were stored under aerobic (AP) or anaerobic (VP) conditions. Vacuum packaging was performed using sous-vide vacuum pouches 20 cm wide by 15 cm long, which were evacuated and closed using a vacuum-sealing machine (Henkelman, ’s-Hertogenboschm, The Netherlands). Both the AP and VP products were kept at 4 and 10 °C for 0, 1, 4 and 7 days.

### 2.5. Microbial Enumeration

After storage, individual samples were aseptically transferred into sterile stomacher bags containing 90 mL peptone water (Himedia, Mumbai, India). Homogenization of the samples was conducted using a stomacher (Interscience, Saint Nom la Brétèche, France) for 1 min. After that, 0.1 or 1 mL of appropriate decimal dilutions was plated in duplicate for Mesophilic total plate count (TPC), yeast and molds (Y&M), mesophilic LAB, and *Enterobacteriaceae* (EN) via the pour plate method using Plate Count Agar (30 °C for 3 day), Sabaroud Dextrose Agar (SDA, 25 °C for 5 day), De Man Rogosa Sharpe Agar (MRS, with an overlay, anaerobically, 25 °C for 5 day), and Violet Red Bile Glucose Agar (VRBGA, with an overlay at 32 °C for 24–48 h), respectively. A spread plate method was used to enumerate pseudomonads using Pseudomonas Agar supplemented with Pseudomonas CFC, and incubated at 25 °C for 48 h. Subsequently, plates containing 25–250 colony-forming units (CFU) were counted manually using a Stuart^®^ Colony Counter (Cole-Parmer, Eaton Socon, UK) and the resulting data were transformed into log CFU/g [17].

### 2.6. Statistical Analysis

Two-factor ANOVA including post-hoc analysis (Tukey HSD) was performed to analyze the effects of EO treatments (fixed factor, 8 levels), storage period (fixed factor, 4 levels,) and their interactive effects on the survival of spoilage-causing microorganisms in the yogurt-based marinade [15]. Data were checked for normality via the Shapiro–Wilk test before ANOVA analysis. In addition, an independent T-test was performed to compare aerobic and vacuum packaging. Statistical difference was tested at (*p* < 0.05). Statistical analysis was carried out using IBM SPSS Statistics (version 26) software. Graphical representations of the analyzed data were prepared using GraphPad Prism Version 7.0 (Graph Pad Software, Inc., La Jolla, CA, USA) [15]. However, the data here are presented in tabular form only.

## 3. Results

The effects of marinades with the added active EOs on the indigenous microbiota of CM during 4 and 10 °C storage for up to 7 d are presented in Table 1, Table 2, Table 3, Table 4, Table 5, Table 6, Table 7, Table 8, Table 9 and Table 10.

For CM stored aerobically, the increases at day 7 in TPC, Y&M, LAB, EN, and PS at 4 °C were 3.1, 4.2, 1.6, 3.9, and 3.9, log CFU/g, respectively, and at 10 °C they were 3.7, 4.5, 3.2, 4.5, and 5.0, log CFU/g, respectively. For MCM, the increases were similar at 4 °C, but for PS it was 2.6 log CFU/g, and at 10 °C they were 2.1, 3.2, 2.4, 0.9, and 1.5 log CFU/g, respectively. When CM was VP-treated and stored at 4 and 10 °C, and when MCM was VP-treated and stored at 4 °C, there was more effective (*p* < 0.05) control of spoilage-causing microorganisms than when AP was used.

On day 7 under AP, the microbial numbers in CM for TPC, Y&M, LAB, and EN at 10 °C were higher than at 4 °C by 0.7, 0.3, 1.6, and 0.7 log CFU/g, and in MCM they were also higher by 1.7, 3.0, 1.5, and 1.0 log CFU/g, respectively. In contrast, PS numbers were 1.1 log CFU/g higher in CM at 4 °C compared to 10 °C.

When EOs were added to MCM and stored aerobically at 4 °C, the maximum decreases in TPC, Y&M, LAB, EB and PS populations after 7 days were 1.2 log CFU/g (2% TH), 1.0 log CFU/g (2% CI), 1.5 log CFU/g (2% CA), 3.1 log CFU/g (2% CI) and 3.5 log CFU/g (2% CI), respectively. The maximum decreases in TPC, Y&M, LAB, EB and PS in the samples stored at 10 °C by 7 day were 0.8 log CFU/g (1% CI), 1.4 log CFU/g (2% TH), 2.1 log CFU/g (1% CI), 3.1 log CFU/g (2% CI) and 4.8 log CFU/g (2% CI), respectively. The maximum decrease in spoilage-causing microorganisms due to the EOs tested at 7 day was significantly (*p* < 0.05) greater than in MCM samples.

Adding EOs to MCM stored under vacuum at 4 °C for 7 days resulted in maximum reductions in TPC, Y&M, LAB, EB and PS populations of 0.4 log CFU/g (2% TH), 0.9 log CFU/g (2% CA), 1.2 log CFU/g (2%TH), 1.4 log CFU/g (2% CI, 2% TH), and 1.2 log CFU/g (2% CI), respectively. When EOs were added to MCM stored under vacuum at 10 °C for 7 day, it resulted in maximum reductions in TPC, LAB, EB and PS populations of 0.8 log CFU/g (2% CA), 0.5 log CFU/g (2% CI), 1.2 log CFU/g (2% TH), and 1.6 log CFU/g (2% CA), respectively. The maximum reduction in spoilage-causing microorganisms due to the EOs tested at 7 day was significantly (*p* < 0.05) greater than in MCM samples. However, EOs had no significant effect (*p* > 0.05) on Y&M populations at 7-day storage under vacuum.

When AP and VP treatments were compared after the addition of EOs, the decrease in microbial populations recovered with Y&M, LAB, EN, and PS media towards the end of the 7-d storage period was the highest under AP at 10 °C, in contrast to the control.

## 4. Discussion

Camel meat demand is growing in the marketplace because it is perceived to be a healthy alternative based on its greater content of vitamins, minerals and amino acids compared to other red meats [4]. A previous study indicated that EOs are an effective means to destroy pathogenic bacteria in camel meat [15]. The EOs and their concentrations used in this study were evaluated in a previous published study, where it was observed that the EOs did not cause any significant changes in color and texture [15]. Amongst the three EOs used in the study (namely, Ca, Ci a nd Th), the greatest scores in terms of color, taste, texture, flavor and overall acceptability were found when Ci (1 or 2%) was added. The lowest scores in terms of taste, flavor and overall acceptance were observed with a Ca substitution. Overall, the study reported a fair overall acceptability of the EO marinated meat when compared to the control. Thereby, the current study has attempted to characterize any interactive inhibitory effects of vacuum packaging and EO treatment on the spoilage-causing microorganisms of marinated camel meat, in order to improve its shelf-life.

In the present study, storing camel meat without (CM) or with marinade (MCM) up to 7 days aerobically at both 4 and 10 °C increased the numbers of spoilage-causing microorganisms. The increase in microbial populations at 4 and 10 °C in response to AP has been recorded previously; the total viable count (TVC)/TPC in beef and sausages under AP were reported to increase by 1.2–5.3 log CFU/g at 4 °C and 4.1 log CFU/g at 10 °C upon storage for 6–7 day, respectively [18,19,20]. The populations of Y&M increased by 2.1–2.4 log CFU/g at 4 °C when stored for about a week in beef and pork, respectively [18,21]. Meanwhile, LAB under AP was reported to increase by 1.2–4.3 log CFU/g at 4 °C, and by 3.9 log CFU/g at 10 °C, upon storage for about a week [18,19,20,21]. Furthermore, the populations of EN increased by 2.2–4.1 log CFU/g at 4 °C in beef [18,20], while the population of PS under AP increased by 3.8 log CFU/g at 4 °C by 7 days [18].

It was observed in the present study that the microbial population increase towards the end of aerobic storage was higher at 10 °C than at 4 °C (except for PS in MCM). The improved microbial growth at the higher of the two temperatures used here has been reported previously. TPC, Y&M, LAB, EN and PS were higher in ground camel meat stored at 10 °C for 7 days compared to 4 °C by 2.4, 1.9, 0.4, 1.1, and 1.8 log CFU/g, respectively [17]. Similarly, in Greek taverna sausages stored up to 6 d, the TVC and LAB populations at 10 °C were higher by 2.9 and 2.7 log CFU/g in the AP product, while they were higher by 2.4 and 2.6 log CFU/g in the VP product, respectively [19]. Another study reported that the total aerobic numbers and the LAB populations in sausages stored at 10 °C were higher by 3 log CFU/g compared to 4 °C after 14 d storage [22].

In the current study, VP was comparatively more effective than AP in retarding bacterial growth in CM and MCM samples. This is understandable as VP reduces oxygen availability, which is vital to the growth of strictly aerobic microorganisms, and its absence slows the growth of facultative organisms. It has been shown that TVC increased in red meat by 1.4–5.3 log CFU/g when held near 4° C, and by 4.3 log CFU/g at 10 °C, when stored for about a week under vacuum [18,19,20,23]. Y&M in VP beef was observed to increase by 0.6 log CFU/g at 4° C by day 7 [18], and in pork it reached 2.9 log CFU/g at 8 d [24]. Similarly, the LAB in red meat increased by 1.6–3.4 log CFU/g at 4 °C and by 4.3 log CFU/g at 10 °C when stored up to a week [18,19,20,23,24]. The EN increased by 0.4–3.3 log CFU/g when meat was stored at 3–4 °C under VP for a week [18,20,23], while the PS increased by 0.9 log CFU/g after storage for 7 days in VP beef [18].

The antimicrobial effects of EOs are ascribed to their ability to disrupt the cell wall/membrane, inhibit adenosine triphosphate (ATP) production, interrupt protein synthesis and unbalance intracellular pH [25]. It is the phenolic compounds in EOs that mainly exert an antimicrobial effect [26].

The antimicrobial effect observed in the current study is similar to observations made under aerobic conditions, where the addition of 0.3–0.5% CA and TH was seen to reduce the aerobic plate count in marinated beef stored at 4 °C for 7 days [27]. Likewise, adding 1% CI, oregano and thyme EOs to pork stored for 6 d at 4 °C decreased Y&M by 2.6, 1.1, and 1.1 log CFU/g, respectively [21]. A similar observation was made in another study [28]. Thyme EO at 1% in pork stored for 6 d at 4 °C decreased LAB numbers by 0.1 log CFU/g [21]. Decreases in PS in beef upon the addition of 0.4 and 0.8% TH and CA have also been reported [28].

Similarly, when the addition of EOs to MCM was followed by VP, there was an additional antimicrobial effect in the current study. A previous study found that when VP pork had been treated with 0.9% TH and stored for 6 d at 3 °C, the TVC numbers were maintained [23]. Similarly, when ham slices were sprayed with mixed rosemary/liquorice extract, vacuum-packaged, and stored at 4 °C for 28 days, the mesophilic aerobic bacteria (MAB) numbers decreased by 3.2 log [29]. Adding oregano decreased LAB by 2.5 log CFU/g, and when vacuum-packed, decreased PS by 6 log CFU/g in beef stored at 5 °C [30]. Likewise, adding 0.9% thyme EO to pork stored at 3 °C for 6 d maintained EB numbers [23].

When the AP and VP results from the present study are compared, the effectiveness of the EOs is seen to be higher under AP conditions in almost all treatments examined. A similar observation has been made previously [28]. This is contrary to the expectation of an additive antimicrobial interaction of VP with EOs. VP has been reported to result in higher drip losses compared to an AP system with beef [31]. It is thought that the increased moisture developing from the meat exudate would have diluted the antimicrobial action of EOs.

Additionally, it was anticipated that there would be an additive or synergistic inhibitory interaction between the use of 4 °C and the EOs. The maximum decreases in Y&M, LAB, EN, and PS were observed when the meat containing EOs was stored at 10 °C. This can be explained by the higher membrane fluidity observed at higher temperatures [16], thereby facilitating greater microbial disruption by the more volatile EOs. Overall, the maximum decreases in TPC, Y&M, LAB, EN, and PS were 1.2, 1.4, 2.1, 3.1 and 4.8 log CFU/g, respectively, based on the treatment conditions.

Meat is sometimes kept briefly at room temperature to accelerate thawing. Further, the retail refrigerators where marinated meat is stored might not always be able to maintain the mandated refrigeration temperatures due to repeated employee entry/exit. Adding EOs would aid in protecting meat under such temperature abuse from spoilage. Additionally, since EOs would be relatively easy to apply at a commercial level and would not require significant equipment investment, transition to their adoption could be rapid.

## 5. Conclusions

These findings illustrate that a greater antimicrobial effectiveness of EOs on marinated camel meat may be anticipated during mild temperature abuse, which can periodically be observed on a practical basis. Antimicrobial effects were enhanced in the presence of EOs if the meat was stored aerobically, rather than under vacuum. Storing MCM with added EOs aerobically at 10 °C was observed to deter spoilage-causing microorganisms to the greatest extent.

## Figures and Tables

**Table 1:** Population changes of mesophilic total plate count (log (N0/N) ± SD CFU/g) in marinated camel meat with essential oils under aerobic (AC) and vacuum (VC) conditions after storage at 4 °C for 0, 1, 4 and 7 days.

TRT	Days	CM	MCM	MCM + 1%Ca	MCM + 2%Ca	MCM + 1%Ci	MCM + 2%Ci	MCM + 1%Th	MCM + 2%Th	*p*-Value
AC	0	0.00 ^cde^	0.00 ^cde^	0.00 ^cde^	0.00 ^cde^	0.00 ^cde^	0.00 ^cde^	0.00 ^cde^	0.00 ^cde^	0.00
	1	0.12 ^cdA^ ± 0.3	−0.17 ^cdefgh^ ± 0.6	−0.44 ^defgh^ ± 0.5	−1.11 ^hiA^ ± 0.2	−0.63 ^efghi^ ± 0.5	−0.86 ^ghi^ ± 0.35	−0.77 ^fghi^ ± 0.2	−0.8 ^ghiA^ ± 0.2	
	4	1.68 ^bA^ ± 0.6	−0.08 ^cdef^ ± 0.6	−0.61 ^efghi^ ± 0.3	−0.75 ^fghiA^ ± 0.1	−0.77 ^fghiA^ ± 0.5	−0.85 ^ghi^ ± 0.2	−0.7 ^efghiA^ ± 0.2	−0.93 ^hiA^ ± 0.1	
	7	3.05 ^aA^ ± 0.2	0.40 ^c^ ± 1.1	−0.7 ^efghiA^ ± 0.3	−1.12 ^hiA^ ± 0.4	−0.64 ^efghBi^ ± 0.4	−1.03 ^hiA^ ± 0.2	−0.72 ^fghiA^ ± 0.2	−1.18 ^iA^ ± 0.2	
VC	0	0.00 ^bcde^	0.00 ^abcd^	0.00 ^abcd^	0.00 ^abcd^	0.00 ^abcd^	0.00 ^abcd^	0.00 ^abcd^	0.00 ^abcd^	0.00
	1	−1.03 ^hB^ ± 0.6	0.33 ^a^ ± 0.1	−0.51 ^efghi^ ± 0.3	−0.67 ^ghiB^ ± 0.3	−0.42 ^efgh^ ± 0.4	−0.49 ^efgh^ ± 0.4	−0.54 ^fghi^ ± 0.5	−0.48 ^efghB^ ± 0.4	
	4	−0.72 ^ghB^ ± 0.5	0.25 ^abc^ ± 0.3	−0.32 ^defgh^ ± 0.1	−0.49 ^fghB^ ± 0.2	−0.37 ^cdefghA^ ± 0.3	−0.37 ^efghB^ ± 0.3	−0.17 ^bcdefgB^ ± 0.3	−0.45 ^efghB^ ± 0.3	
	7	0.39 ^aB^ ± 0.5	0.2 ^abcd^ ± 0.3	−0.13 ^abcdefB^ ± 0.4	−0.26 ^cdefghB^ ± 0.3	−0.19 ^abcdefB^ ± 0.3	−0.19 ^bcdefghB^ ± 0.3	−0.08 ^abcdefB^ ± 0.2	−0.4 ^efgh^ ± 0.4	
AC X VC	0	NS	NS	NS	NS	NS	NS	NS	NS	
	1	<0.001	NS	NS	0.002	NS	NS	NS	NS	
	4	<0.0001	NS	NS	0.0011	0.00	<0.0001	<0.0001	<0.0001	
	7	<0.0001	NS	0.002	<0.001	0.003	<0.0001	<0.0001	<0.0001	

a–i Different letters indicate significant differences between days and the treatments (*p* < 0.05). A,B Populations under the same treatments in each column followed by different letters are significantly different (*p* < 0.05).—Initial quantity (N0) of camel meat (CM) in aerobic and vacuum packaging: 5.4 ± 0.2 and 5.0 ± 0.5 log CFU/g; marinated camel meat (MCM) in aerobic and vacuum packaging: 5.1 ± 0.4 and 6.0 ± 0.2 CFU/g.—MCM + 1% Ca: marinated camel meat and 1% carvacrol; MCM + 2% Ca: marinated camel meat and 2% carvacrol; MCM + 1% Ci: marinated camel meat and 1% cinnamaldehyde; MCM + 2% Ci: marinated camel meat and 2% cinnamaldehyde; MCM + 1% Th: marinated camel meat and 1% thymol; MCM + 2% Th: marinated camel meat and 2% thymol. Significantly different at (*p* < 0.05), NS—non-significant.

**Table 2:** Population changes of mesophilic total plate count (log (N0/N) ± SD CFU/g) in marinated camel meat with essential oils under aerobic (AC) and vacuum (VC) conditions after storage at 10 °C for 0, 1, 4 and 7 days.

TRT	Days	CM	MCM	MCM + 1%Ca	MCM + 2%Ca	MCM + 1%Ci	MCM + 2%Ci	MCM + 1%Th	MCM + 2%Th	*p*-Value
AC	0	0.00 ^ef^	0.00 ^ef^	0.00 ^ef^	0.00 ^ef^	0.00 ^ef^	0.00 ^ef^	0.00 ^ef^	0.00 ^ef^	0.00
	1	0.77 ^deA^ ± 0.3	−0.09 ^efA^ ± 0.2	−0.36 ^fA^ ± 0.1	−0.77 ^f^ ± 0.2	−0.59 ^f^ ± 0.2	−0.58 ^f^ ± 0.2	−0.29 ^fA^ ± 0.2	−0.53 ^fA^ ± 0.1	
	4	2.71 ^bA^ ± 0.5	1.32 ^cdA^ ± 0.6	−0.5 ^f^ ± 0.6	−0.64 ^f^ ± 0.5	−0.62 ^fA^ ± 0.2	−0.86 ^fA^ ± 0.1	−0.5 ^f^ ± 0.6	−0.82 ^f^ ± 0.5	
	7	3.71 ^aA^ ± 0.3	2.05 ^bcA^ ± 0.8	−0.32 ^f^ ± 0.8	−0.45 ^f^ ± 0.8	−0.79 ^f^ ± 0.1	−0.6 ^f^ ± 0.4	−0.2 ^ef^ ± 1.0	−0.28 ^f^ ± 1.0	
VC	0	0.00 ^abcd^	0.00 ^abcd^	0.00 ^abcd^	0.00 ^abcd^	0.00 ^abcd^	0.00 ^abcd^	0.00 ^abcd^	0.00 ^abcd^	0.00
	1	−1.16 ^fB^ ± 0.4	0.15 ^bB^ ± 0.3	−0.75 ^efgB^ ± 0.3	−0.8 ^ef^ ± 0.4	−0.6 ^ef^ ± 0.4	−0.83 ^ef^ ± 0.4	−0.81 ^efB^ ± 0.3	−0.81 ^ef^ ± 0.4	
	4	−0.68 ^efgB^ ± 0.6	0.05 ^abc^ ± 0.2	−0.44 ^cdef^ ± 0.2	−0.68 ^efg^ ± 0.3	−0.35 ^bcdef^ ± 0.3	−0.53 ^defB^ ± 0.3	−0.56 ^ef^ ± 0.3	−0.71 ^efg^ ± 0.4	
	7	0.26 ^aB^ ± 0.5	0.01 ^abc^ ± 0.3	−0.23 ^abcde^ ± 0.2	−0.75 ^efg^ ± 0.4	−0.4 ^cdef^ ± 0.3	−0.54 ^ef^ ± 0.3	−0.22 ^abcde^ ± 0.3	−0.61 ^ef^ ± 0.3	
AC X VC	0	NS	NS	NS	NS	NS	NS	NS	NS	
	1	<0.0001	0.024	<0.001	NS	NS	NS	<0.0001	0.026	
	4	<0.0001	<0.0001	NS	NS	<0.010	<0.001	NS	NS	
	7	<0.0001	<0.0001	NS	NS	<0.0001	NS	NS	NS	

a–g Different letters indicate significant differences between days and the treatments (*p* < 0.05). A,B Populations under the same treatments in each column followed by different letters are significantly different (*p* < 0.05).—Initial quantity (N0) of camel meat (CM) in aerobic and vacuum packaging: 4.9 ± 0.3 and 5.2 ± 0.1 log CFU/g; marinated camel meat (MCM) in aerobic and vacuum packaging: 4.7 ± 0.6 and 6.1 ± 0.3 CFU/g.—MCM + 1% Ca: marinated camel meat and 1% carvacrol; MCM + 2% Ca: marinated camel meat and 2% carvacrol; MCM + 1% Ci: marinated camel meat and 1% cinnamaldehyde; MCM + 2% Ci: marinated camel meat and 2% cinnamaldehyde; MCM + 1% Th: marinated camel meat and 1% thymol; MCM + 2% Th: marinated camel meat and 2% thymol. Significantly different at (*p* < 0.05), NS—non-significant.

**Table 3:** Population changes of yeast and molds (log (N0/N) ± SD CFU/g) in marinated camel meat with essential oils under (AC) aerobic and (VC) vacuum conditions after storage at 4 °C for 0, 1, 4 and 7 days.

TRT	Days	CM	MCM	MCM + 1%Ca	MCM + 2%Ca	MCM + 1%Ci	MCM + 2%Ci	MCM + 1%Th	MCM + 2%Th	*p*-Value
AC	0	0.00 ^defg^	0.00 ^defg^	0.00 ^defg^	0.00 ^defg^	0.00 ^defg^	0.00 ^defg^	0.00 ^defg^	0.00 ^defg^	0.00
	1	1.24 ^bcA^ ± 0.4	0.55 ^cd^ ± 0.4	0.25 ^deA^ ± 0.2	−0.71 ^fghA^ ± 0.2	−0.01 ^defgA^ ± 0.4	−0.25 ^defgh^ ± 0.2	0.05 ^defA^ ± 0.4	−0.46 ^efghA^ ± 0.4	
	4	1.28 ^bcA^ ± 0.2	1.27 ^bc^ ± 0.7	1.13 ^bcA^ ± 0.8	−0.77 ^gh^ ± 0.6	0.00 ^defg^ ± 1.1	−0.45 ^efgh^ ± 0.7	−0.68 ^fgh^ ± 0.4	−0.84 ^h^ ± 0.4	
	7	4.2 ^aA^ ± 0.2	0.23 ^de^ ± 0.5	−0.58 ^fgh^ ± 0.4	−0.59 ^fgh^ ± 0.5	−0.6 ^fgh^ ± 0.5	−0.98 ^h^ ± 0.4	−0.75 ^fgh^ ± 0.4	−0.87 ^h^ ± 0.3	
VC	0	0 ^abcd^	0.00 ^abcd^	0.00 ^abcd^	0.00 ^abcd^	0.00 ^abcd^	0.00 ^abcd^	0.00 ^abcd^	0.00 ^abcd^	0.00
	1	−0.67 ^cdeB^ ± 0.7	0.08 ^abc^ ± 0.5	−0.35 ^bcdB^ ± 0.3	−0.95 ^cdeB^ ± 0.2	−0.7 ^cdB^ ± 0.8	−0.38 ^bcd^ ± 0.8	−0.92 ^cdeB^ ± 0.7	−0.89 ^cdeB^ ± 0.3	
	4	0.14 ^abcB^ ± 0.6	0.98 ^a^ ± 0.4	−0.41 ^bcdB^ ± 0.7	−0.61 ^cd^ ± 0.9	−0.46 ^cd^ ± 0.6	−0.91 ^cde^ ± 0.5	−0.8 ^cde^ ± 0.4	−1.15 ^de^ ± 0.5	
	7	0.74 ^abB^ ± 0.7	0.02 ^abcd^ ± 0.5	−0.4 ^bcd^ ± 0.6	−0.92 ^cde^ ± 0.7	−0.62 ^cd^ ± 0.6	−0.8 ^cde^ ± 0.6	−0.45 ^cd^ ± 0.5	−0.78 ^cde^ ± 0.8	
AC X VC	0	NS	NS	NS	NS	NS	NS	NS	NS	
	1	0.008	NS	<0.006	0.043	<0.024	NS	<0.001	0.014	
	4	<0.0001	NS	<0.0001	NS	NS	NS	NS	NS	
	7	<0.0001	NS	NS	NS	NS	NS	NS	NS	

a–h Different letters indicate significant differences between days and the treatments (*p* < 0.05). A,B Populations under the same treatments in each column followed by different letters are significantly different (*p* < 0.05).—Initial quantity (N0) of camel meat (CM) in aerobic and vacuum packaging: 4.9 ± 0.3 and 5.2 ± 0.1 log CFU/g; marinated camel meat (MCM) in aerobic and vacuum packaging: 4.7 ± 0.6 and 6.1 ± 0.3 CFU/g.—MCM + 1% Ca: marinated camel meat and 1% carvacrol; MCM + 2% Ca: marinated camel meat and 2% carvacrol; MCM + 1% Ci: marinated camel meat and 1% cinnamaldehyde; MCM + 2% Ci: marinated camel meat and 2% cinnamaldehyde; MCM + 1% Th: marinated camel meat and 1% thymol; MCM + 2% Th: marinated camel meat and 2% thymol. Significantly different at (*p* < 0.05), NS—non-significant.

**Table 4:** Population changes of yeast and molds (log (N0/N) ± SD CFU/g) in marinated camel meat with essential oils under aerobic (AC) and vacuum (VC) conditions after storage at 10 °C for 0, 1, 4 and 7 days.

TRT	Days	CM	MCM	MCM+1%Ca	MCM+2%Ca	MCM+1%Ci	MCM+2%Ci	MCM+1%Th	MCM+2%Th	*p*-Value
AC	0	0.00 ^cdef^	0.00 ^cdef^	0.00 ^cdef^	0.00 ^cdef^	0.00 ^cdef^	0.00 ^cdef^	0.00 ^cdef^	0.00 ^cdef^	0.00
	1	0.5 ^cdA^ ± 0.1	−0.69 ^def^ ± 0.2	−0.55 ^cdefA^ ± 0.2	−1.47 ^f^ ± 0.7	−1.29 ^ef^ ± 0.4	−1.51 ^f^ ± 0.2	−0.95 ^defA^ ± 0.2	−1.29 ^efA^ ± 0.9	
	4	4.58 ^aA^ ± 0.4	1.03 ^bcA^ ± 0.5	0.34 ^cdeA^ ± 0.5	−0.84 ^def^ ± 0.5	−0.49 ^cdef^ ± 0.7	−1.16 ^def^ ± 0.6	−0.37 ^cdef^ ± 0.9	−1.1 ^f^ ± 0.9	
	7	4.48 ^aA^ ± 0.5	3.19 ^b^ ± 0.2	0.26 ^cdefA^ ± 0.5	−1.17 ^defA^ ± 1.0	−0.4 ^cdefA^ ± 0.8	−0.63 ^defA^ ± 0.3	−0.51 ^cdefA^ ± 1.0	−1.44 ^defA^ ± 0.7	
VC	0	0.00 ^bcdef^	0.00 ^bcdef^	0.00 ^bcdef^	0.00 ^bcdef^	0.00 ^bcdef^	0.00 ^bcdef^	0.00 ^bcdef^	0.00 ^bcdef^	0.00
	1	−0.93 ^fghiB^ ± 0.1	0.2 ^abcde^ ± 1.2	−1.37 ^ghiB^ ± 0.3	−1.07 ^ghi^ ± 0.3	−1.5 ^ghi^ ± 0.4	−1.51 ^ghi^ ± 0.4	−1.84 ^iB^ ± 0.6	−1.81 ^hiB^ ± 0.4	
	4	−0.09 ^bcdefB^ ± 0.5	−0.17 ^cdefgB^ ± 0.6	−0.86 ^fghiB^ ± 0.6	−0.18 ^cdefgh^ ± 0.3	−0.31 ^defgh^ ± 0.6	−0.63 ^efghi^ ± 0.5	−0.19 ^cdefgh^ ± 0.4	−0.44 ^efgh^ ± 0.4	
	7	0.17 ^abcdeB^ ± 0.6	0.97 ^a^ ± 0.5	0.62 ^abcB^ ± 0.3	0.14 ^abcdeB^ ± 0.5	0.63 ^abcB^ ± 0.6	0.54 ^abcdB^ ± 0.5	0.76 ^abB^ ± 0.4	0.56 ^abcdB^ ± 0.4	
AC X VC	0	NS	NS	NS	NS	NS	NS	NS	NS	
	1	<0.0001	NS	0.001	NS	NS	NS	<0.001	<0.0001	
	4	<0.0001	0.003	<0.0001	NS	NS	NS	NS	0.05	
	7	<0.0001	NS	0.043	0.015	<0.09	<0.0001	0.002	0.02	

a–i Different letters indicate significant differences between days and the treatments (*p* < 0.05). A,B Populations under the same treatments in each column followed by different letters are significantly different (*p* < 0.05).—Initial quantity (N0) of camel meat (CM) in aerobic and vacuum packaging: 4.9 ± 0.3 and 5.2 ± 0.1 log CFU/g; marinated camel meat (MCM) in aerobic and vacuum packaging: 4.7 ± 0.6 and 6.1 ± 0.3 CFU/g.—MCM + 1% Ca: marinated camel meat and 1% carvacrol; MCM + 2% Ca: marinated camel meat and 2% carvacrol; MCM + 1% Ci: marinated camel meat and 1% cinnamaldehyde; MCM + 2% Ci: marinated camel meat and 2% cinnamaldehyde; MCM + 1% Th: marinated camel meat and 1% thymol; MCM + 2% Th: marinated camel meat and 2% thymol. Significantly different at (*p* < 0.05), NS—non-significant.

**Table 5:** Population changes of mesophilic lactic acid bacteria (log (N0/N) ± SD CFU/g) in marinated camel meat with essential oils under aerobic (AC) and vacuum (VC) conditions after storage at 4 °C for 0, 1, 4 and 7 days.

TRT	Days	CM	MCM	MCM + 1%Ca	MCM + 2%Ca	MCM + 1%Ci	MCM + 2%Ci	MCM + 1%Th	MCM + 2%Th	*p*-Value
AC	0	0.00 ^bcde^	0.00 ^bcde^	0.00 ^bcde^	0.00 ^bcde^	0.00 ^bcde^	0.00 ^bcde^	0.00 ^bcde^	0.00 ^bcde^	0.00
	1	−0.15 ^bcdef^ ± 0.6	−0.5 ^defgh^ ± 0.8	−0.03 ^bcde^ ± 0.5	−0.83 ^defghA^ ± 0.3	0.11 ^bcd^ ± 0.4	−1.1 ^efghA^ ± 0.8	−0.40 ^cdefghA^ ± 0.5	−0.97 ^defghA^ ± 0.4	
	4	−0.05 ^bcdeA^ ± 0.7	−0.39 ^cdefghA^ ± 0.6	−1.01 ^defghA^ ± 0.7	−0.77 ^defgh^ ± 0.3	−0.75 ^defghA^ ± 0.7	−1.13 ^efghA^ ± 0.6	−0.60 ^defgh^ ± 0.3	−1.36 ^ghA^ ± 0.2	
	7	1.6 ^aA^ ± 0.7	0.93 ^abA^ ± 0.9	0.69 ^abcA^ ± 0.5	−1.51 ^hA^ ± 0.6	−0.58 ^defghA^ ± 0.7	−0.36 ^cdefgA^ ± 0.4	−0.06 ^bcdef^ ± 0.2	−1.18 ^fgh^ ± 0.0	
VC	0	0.00 ^abc^	0.00 ^abc^	0.00 ^abc^	0.00 ^abc^	0.00 ^abc^	0.00 ^abc^	0.00 ^abc^	0.00 ^abc^	0.00
	1	−0.07 ^abc^ ± 0.1	0.34 ^ab^ ± 0.4	0.35 ^ab^ ± 0.4	0.06 ^abcB^ ± 0.3	0.52 ^ab^ ± 0. 6	0.09 ^abB^ ± 0.3	0.37 ^abB^ ± 0.4	−0.06 ^abcB^ ± 0.4	
	4	−0.93 ^cdeB^ ± 0.4	0.51 ^abB^ ± 0.6	0.67 ^aB^ ± 0.2	−0.41 ^abcde^ ± 0.7	0.47 ^abB^ ± 0.4	−0.21 ^abcdeB^ ± 0.3	−0.14 ^abcd^ ± 0.7	−0.14 ^abcdB^ ± 0.7	
	7	−1.25 ^cB^ ± 0.2	−0.51 ^bcdeB^ ± 0.7	−0.51 ^bcdeB^ ± 0.6	−0.76 ^cdeB^ ± 0.7	0.08 ^abcB^ ± 0.5	−0.87 ^cdeB^ ± 0.8	−0.18 ^abcde^ ± 0.2	−1.23 ^de^ ± 0.6	
AC X VC	0	NS	NS	0.013	0.013	0.013	0.013	0.013	0.013	
	1	NS	NS	NS	<0.0001	NS	0.003	0.009	0.001	
	4	<0.03	0.002	<0.0001	NS	<0.0001	0.004	NS	0.001	
	7	<0.0001	<0.005	0.001	<0.05	<0.05	0.008	NS	NS	

a–h Different letters indicate significant differences between days and the treatments (*p* < 0.05). A,B Populations under the same treatments in each column followed by different letters are significantly different (*p* < 0.05).—Initial quantity (N0) of camel meat (CM) in aerobic and vacuum packaging: 4.9 ± 0.3 and 5.2 ± 0.1 log CFU/g; marinated camel meat (MCM) in aerobic and vacuum packaging: 4.7 ± 0.6 and 6.1 ± 0.3 CFU/g.—MCM + 1% Ca: marinated camel meat and 1% carvacrol; MCM + 2% Ca: marinated camel meat and 2% carvacrol; MCM + 1% Ci: marinated camel meat and 1% cinnamaldehyde; MCM + 2% Ci: marinated camel meat and 2% cinnamaldehyde; MCM + 1% Th: marinated camel meat and 1% thymol; MCM + 2% Th: marinated camel meat and 2% thymol. Significantly different at (*p* < 0.05), NS—non-significant.

**Table 6:** Population changes of mesophilic lactic acid bacteria (log (N0/N) ± SD CFU/g) in marinated camel meat with essential oils under aerobic (AC) and vacuum (VC) conditions after storage at 10 °C for 0, 1, 4 and 7 day.

TRT	Days	CM	MCM	MCM + 1%Ca	MCM + 2%Ca	MCM + 1%Ci	MCM + 2%Ci	MCM + 1%Th	MCM + 2%Th	*p*-Value
AC	0	0.00 ^def^	0.00 ^def^	0.00 ^def^	0.00 ^def^	0.00 ^def^	0.00 ^def^	0.00 ^def^	0.00 ^def^	0.00
	1	−0.45 ^defghA^ ± 0.6	−0.15 ^defgh^ ± 0.4	−1.01 ^fgi^ ± 0.3	−0.6 ^defgh^ ± 0.8	0.53 ^cdeA^ ± 0.8	−0.31 ^defgA^ ± 0.5	0.04 ^defA^ ± 0.5	−0.35 ^defgh^ ± 0.4	
	4	2.66 ^aA^ ± 0.6	0.35 ^cde^ ± 0.8	−0.41 ^defgh^ ± 0.7	0.38 ^cde^ ± 0.5	−0.91 ^fghi^ ± 0.8	−1.52 ^hiA^ ± 0.4	0.12 ^defA^ ± 0.1	−1.03 ^fghi^ ± 0.9	
	7	3.24 ^aA^ ± 0.3	2.4 ^abA^ ± 0.6	0.72 ^cdA^ ± 0.8	0.3 ^cde^ ± 0.9	−2.06 ^iA^ ± 0.8	−1.28 ^ghiAk^ ± 0.1	1.35 ^bcA^ ± 0.7	1 ^bcAA^ ± 0.7	
VC	0	0.00 ^bcde^	0.00 ^bcde^	0.00 ^bcd^	0.00 ^bcd^	0.00 ^bcd^	0.00 ^bcd^	0.00 ^bcd^	0.00 ^bcd^	0.00
	1	0.16 ^bcdB^ ± 0.5	−0.2 ^bcde^ ± 0.3	−0.63 ^defg^ ± 0.3	−0.47 ^bcdefg^ ± 0.4	−1.13 ^gh^ ± 0.7	−0.89 ^fgB^ ± 0.4	−1.86 ^iB^ ± 0.3	−0.55 ^defg^ ± 0.3	
	4	1.85 ^aB^ ± 0.7	0.07 ^bcd^ ± 0.5	−0.55 ^defg^ ± 0.5	−0.7 ^defg^ ± 0.2	−0.63 ^defg^ ± 0.6	−0.84 ^efgB^ ± 0.7	−0.31 ^bcdefgB^ ± 0.3	−0.51 ^cdefg^ ± 0.6	
	7	2.00 ^aB^ ± 0.4	0.38 ^bB^ ± 0.4	−0.23 ^bcdeB^ ± 0.4	−0.13 ^bcde^ ± 0.3	−0.09 ^bcdeB^ ± 0.5	−0.5 ^cdefgB^ ± 0.4	0.34 ^bcB^ ± 0.1	−0.16 ^abcdeB^ ± 0.2	
AC X VC	0	NS	NS	NS	NS	NS	NS	NS	NS	
	1	<0.0011	NS	NS	NS	0.004	0.03	<0.0001	NS	
	4	<0.003	NS	NS	0.001	NS	0.0008	0.001	NS	
	7	<0.0001	<0.0001	0.02	NS	<0.0001	<0.0001	NS	0.024	

a–i Different letters indicate significant differences between days and the treatments (*p* < 0.05). A,B Populations under the same treatments in each column followed by different letters are significantly different (*p* < 0.05).—Initial quantity (N0) of camel meat (CM) in aerobic and vacuum packaging: 4.9 ± 0.3 and 5.2 ± 0.1 log CFU/g; marinated camel meat (MCM) in aerobic and vacuum packaging: 4.7 ± 0.6 and 6.1 ± 0.3 CFU/g.—MCM + 1% Ca: marinated camel meat and 1% carvacrol; MCM + 2% Ca: marinated camel meat and 2% carvacrol; MCM + 1% Ci: marinated camel meat and 1% cinnamaldehyde; MCM + 2% Ci: marinated camel meat and 2% cinnamaldehyde; MCM + 1% Th: marinated camel meat and 1% thymol; MCM + 2% Th: marinated camel meat and 2% thymol. Significantly different at (*p* < 0.05), NS—non-significant.

**Table 7:** Population changes of *Enterobacteriaceae* (log (N0/N) ± SD CFU/g) in marinated camel meat with essential oils under aerobic (AC) and vacuum (VC) conditions after storage at 4 °C for 0, 1, 4 and 7 days.

TRT	Days	CM	MCM	MCM+1%Ca	MCM+2%Ca	MCM+1%Ci	MCM+2%Ci	MCM+1%Th	MCM+2%Th	*p*-Value
AC	0	0.00 ^cde^	0.00 ^cde^	0.00 ^cde^	0.00 ^cde^	0.00 ^cde^	0.00 ^cde^	0.00 ^cde^	0.00 ^cde^	0.00
	1	0.46 ^bcA^ ± 0.2	0.33 ^bcd^ ± 0.5	−0.52 ^cdefgA^ ± 0.3	−0.77 ^cdefghA^ ± 0.2	−1.08 ^efgh^ ± 0.7	−0.95 ^efgh^ ± 0.2	−0.68 ^cdefghA^ ± 0.2	−1.68 ^fghi^ ± 0.4	
	4	1.44 ^bA^ ± 0.6	0.31 ^bcdA^ ± 0.3	−1.24 ^efghi^ ± 1.0	−1.53 ^fghi^ ± 0.9	−1.96 ^hij^ ± 0.8	−1.81 ^hij^ ± 1.1	−1.34 ^efghiA^ ± 0.2	−2.43 ^ijA^ ± 0.4	
	7	3.88 ^aA^ ± 0.2	−0.15 ^cdeA^ ± 0.4	−0.40 ^cd^ ± 0.3	−2.51 ^ijA^ ± 0.1	−1.68 ^fghiA^ ± 0.4	−3.05 ^jA^ ± 0.3	−0.93 ^defgh^ ± 0.6	−2.15 ^ij^ ± 0.6	
VC	0	0.00 ^abc^	0.00 ^abc^	0.00 ^abc^	0.00 ^abc^	0.00 ^abc^	0.00 ^abc^	0.00 ^abc^	0.00 ^abc^	0.00
	1	−0.81 ^cdefB^ ± 0.8	−0.27 ^abcd^ ± 0.8	−1.91 ^fB^ ± 0.2	−1.88 ^fB^ ± 0.4	−0.23 ^abcd^ ± 0.8	−1.91 ^f^ ± 0.1	0.15 ^abcB^ ± 0.0	−1.84 ^f^ ± 0.3	
	4	−0.39 ^bcdeB^ ± 0.9	−0.85 ^cdefB^ ± 0.2	−0.72 ^cdef^ ± 0.5	−1.58 ^ef^ ± 0.4	−0.02 ^abc^ ± 0.8	−0.97 ^cdef^ ± 0.4	−0.54 ^bcdeB^ ± 0.4	−1.48 ^efB^ ± 0.6	
	7	0.93 ^aB^ ± 0.4	0.63 ^abB^ ± 0.6	−0.15 ^abc^ ± 0.7	−0.2 ^abcB^ ± 0.6	−0.85 ^cdefB^ ± 0.6	−1.39 ^fB^ ± 0.5	−0.54 ^bcde^ ± 0.2	−1.42 ^def^ ± 0.5	
AC X VC	0	NS	NS	NS	NS	NS	NS	NS	NS	
	1	<0.003	NS	<0.0001	0.01	NS	NS	0.001	NS	
	4	<0.0001	<0.0001	NS	NS	NS	NS	0.04	0.027	
	7	<0.0001	0.025	NS	<0.0001	0.04	0.03	NS	NS	

a–j Different letters indicate significant differences between days and the treatments (*p* < 0.05). A,B Populations under the same treatments in each column followed by different letters are significantly different (*p* < 0.05).—Initial quantity (N0) of camel meat (CM) in aerobic and vacuum packaging: 4.9 ± 0.3 and 5.2 ± 0.1 log CFU/g; marinated camel meat (MCM) in aerobic and vacuum packaging: 4.7 ± 0.6 and 6.1 ± 0.3 CFU/g.—MCM + 1% Ca: marinated camel meat and 1% carvacrol; MCM + 2% Ca: marinated camel meat and 2% carvacrol; MCM + 1% Ci: marinated camel meat and 1% cinnamaldehyde; MCM + 2% Ci: marinated camel meat and 2% cinnamaldehyde; MCM + 1% Th: marinated camel meat and 1% thymol; MCM + 2% Th: marinated camel meat and 2% thymol. Significantly different at (*p* < 0.05), NS—non-significant.

**Table 8:** Population changes of *Enterobacteriaceae* (log (N0/N) ± SD CFU/g) in marinated camel meat with essential oils under aerobic (AC) and vacuum (VC) conditions after storage at 10 °C for 0, 1, 4 and 7 days.

TRT	Days	CM	MCM	MCM + 1%Ca	MCM + 2%Ca	MCM + 1%Ci	MCM + 2%Ci	MCM + 1%Th	MCM + 2%Th	*p*-Value
AC	0	0.00 ^bcde^	0.00 ^bcde^	0.00 ^bcde^	0.00 ^bcde^	0.00 ^bcde^	0.00 ^bcde^	0.00 ^bcde^	0.00 ^bcde^	0.00
	1	0.98 ^bA^ ± 0.2	−0.33 ^cdef^ ± 0.6	−1.25 ^efgh^ ± 0.8	−2.10 ^hij^ ± 0.1	−1.49 ^fghi^ ± 0.3	−2.26 ^hij^ ± 0.3	−1.68 ^ghi^ ± 0.8	−2.13 ^hij^ ± 0.2	
	4	4.09 ^aA^ ± 0.3	0.4 ^bc^ ± 0.2	−0.62 ^defgA^ ± 0.1	−1.27 ^efgh^ ± 0.2	−1.89 ^ghijA^ ± 0.1	−2.52 ^hij^ ± 0.4	−1.43 ^fghi^ ± 0.3	−1.75 ^ghi^ ± 0.8	
	7	4.53 ^aA^ ± 0.1	0.89 ^bcA^ ± 0.9	−0.60 ^defg^ ± 0.9	−1.13 ^efgh^ ± 0.9	−2.08 ^hijA^ ± 0.1	−3.06 ^j^ ± 0.1	−1.29 ^efghi^ ± 0.4	−2.6 ^ijA^ ± 0.1	
VC	0	0.00 ^a^	0.00 ^a^	0.00 ^a^	0.00 ^a^	0.00 ^a^	0.00 ^a^	0.00 ^a^	0.00 ^a^	0.00
	1	−0.89 ^abcB^ ± 0.4	−0.32 ^ab^ ± 0.9	−1.04 ^abc^ ± 0.8	−1.46 ^c^ ± 1.0	−1.39 ^bc^ ± 0.7	−1.05 ^abcB^ ± 0.4	−0.84 ^abc^ ± 1.0	−1.09 ^bcB^ ± 0.7	
	4	−0.76 ^abcB^ ± 0.3	−0.66 ^abcde^ ± 0.8	−1.21 ^bcB^ ± 0.4	−0.76 ^abc^ ± 0.7	−0.64 ^abcB^ ± 0.2	−1.01 ^abcB^ ± 0.6	−1.33 ^c^ ± 0.6	−1.51 ^c^ ± 0.5	
	7	−0.33 ^abB^ ± 0.3	−0.55 ^abcB^ ± 0.5	−0.5 ^abc^ ± 0.2	−1.00 ^abc^ ± 0.4	−0.95 ^abcB^ ± 0.7	−0.88 ^abcB^ ± 0.5	−1.03 ^abc^ ± 0.7	−1.17 ^bcB^ ± 0.4	
AC X VC	0	NS	NS	NS	NS	NS	NS	NS	NS	
	1	<0.0001	NS	NS	NS	NS	0.001	NS	0.009	
	4	<0.0001	NS	0.003	NS	<0.0001	0.002	NS	NS	
	7	<0.0001	0.02	NS	NS	<0.0001	0.001	NS	<0.0001	

a–j Different letters indicate significant differences between days and the treatments (*p* < 0.05). A,B Populations under the same treatments in each column followed by different letters are significantly different (*p* < 0.05).—Initial quantity (N0) of camel meat (CM) in aerobic and vacuum packaging: 4.9 ± 0.3 and 5.2 ± 0.1 log CFU/g; marinated camel meat (MCM) in aerobic and vacuum packaging: 4.7 ± 0.6 and 6.1 ± 0.3 CFU/g.—MCM + 1% Ca: marinated camel meat and 1% carvacrol; MCM + 2% Ca: marinated camel meat and 2% carvacrol; MCM + 1% Ci: marinated camel meat and 1% cinnamaldehyde; MCM + 2% Ci: marinated camel meat and 2% cinnamaldehyde; MCM + 1% Th: marinated camel meat and 1% thymol; MCM + 2% Th: marinated camel meat and 2% thymol. Significantly different at (*p* < 0.05), NS—non-significant.

**Table 9:** Population changes of *Pseudomonas* (log (N0/N) ± SD CFU/g) in marinated camel meat with essential oils under aerobic (AC) and vacuum (VC) conditions after storage at 4 °C for 0, 1, 4 and 7 days.

TRT	Days	CM	MCM	MCM + 1%Ca	MCM + 2%Ca	MCM + 1%Ci	MCM + 2%Ci	MCM + 1%Th	MCM + 2%Th	*p*-Value
AC	0	0.00 ^def^	0.00 ^def^	0.00 ^def^	0.00 ^def^	0.00 ^def^	0.00 ^def^	0.00 ^def^	0.00 ^def^	0.00
	1	0.72 ^deA^ ± 0.2	0.57 ^deA^ ± 0.1	0.01 ^defA^ ± 0.4	−2.72 ^jklA^ ± 0.3	−0.07 ^ef^ ± 0.9	−1.25 ^ghi^ ± 0.5	−0.75 ^fg^ ± 0.4	−1.28 f^ghi^ ± 0.7	
	4	1.15 ^cdA^ ± 0.4	2.27 ^bcA^ ± 0.1	−0.75 ^fg^ ± 0.6	−2.11 ^ijkA^ ± 0.4	−1.71 ^ghijA^ ± 0.2	−3.04 ^klA^ ± 0.3	−1.02 ^fghi^ ± 0.2	−1.95 f^hijk^ ± 0.7	
	7	3.9 ^aA^ ± 0.4	2.63 ^bA^ ± 0.1	−0.87 ^fgh^ ± 0.9	−2.01 ^hijk^ ± 0.7	−2.18 ^ijkA^ ± 0.5	−3.54 ^lA^ ± 0.2	−1.04 ^fghi^ ± 0.4	−2.26 ^jklA^ ± 0.7	
VC	0	0.00 ^abc^	0.00 ^abc^	0.00 ^abc^	0.00 ^abc^	0.00 ^abc^	0.00 ^abc^	0.00 ^abc^	0.00 ^abc^	0.00
	1	−0.73 ^bcdeB^ ± 0.6	−0.98 ^cdeB^ ± 0.6	−1.03 ^cdeB^ ± 0.8	−1.14 ^cdeB^ ± 0.6	−1.22 ^de^ ± 0.7	−1.08 ^e^+0.5	−1.1 ^cde^ ± 0.8	−1.04 ^cde^ ± 0.8	
	4	0.51 ^aB^ ± 0.5	−0.33 ^abcdB^ ± 0.6	−1.04 ^cde^ ± 0.6	−1.31 ^deB^ ± 0.6	−0.98 ^cdeB^ ± 0.7	−1.2 ^deB^ ± 0.7	−1.2 ^de^ ± 0.6	−1.42 ^de^ ± 0.3	
	7	−0.32 ^abcdB^ ± 0.7	0.33 ^abB^ ± 0.5	−0.91 ^cde^ ± 0.8	−0.89 ^cde^ ± 0.9	−1.12 ^cdeB^ ± 0.7	−1.19 ^deB^ ± 0.9	−0.78 ^bcde^ ± 0.7	−0.68 ^bcdeB^ ± 0.4	
AC X VC	0	NS	NS	NS	NS	NS	NS	NS	NS	
	1	<0.0001	<0.0001	0.007	0.006	NS	NS	NS	NS	
	4	0.010	<0.0001	NS	0.021	0.013	<0.0001	NS	NS	
	7	<0.0001	<0.0001	NS	0.101	0.018	<0.0001	NS	<0.0001	

a–l Different letters indicate significant differences between days and the treatments (*p* < 0.05). A,B Populations under the same treatments in each column followed by different letters are significantly different (*p* < 0.05).—Initial quantity (N0) of camel meat (CM) in aerobic and vacuum packaging: 4.9 ± 0.3 and 5.2 ± 0.1 log CFU/g; marinated camel meat (MCM) in aerobic and vacuum packaging: 4.7 ± 0.6 and 6.1 ± 0.3 CFU/g.—MCM + 1% Ca: marinated camel meat and 1% carvacrol; MCM + 2% Ca: marinated camel meat and 2% carvacrol; MCM + 1% Ci: marinated camel meat and 1% cinnamaldehyde; MCM + 2% Ci: marinated camel meat and 2% cinnamaldehyde; MCM + 1% Th: marinated camel meat and 1% thymol; MCM + 2% Th: marinated camel meat and 2% thymol. Significantly different at (*p* < 0.05), NS—non-significant.

**Table 10:** Population changes of *Pseudomonas* (log (N0/N) ± SD CFU/g) in marinated camel meat with essential oils under aerobic (AC) and vacuum (VC) conditions after storage at 10 °C for 0, 1, 4 and 7 days.

TRT	Days	CM	MCM	MCM + 1%Ca	MCM + 2%Ca	MCM + 1%Ci	MCM + 2%Ci	MCM + 1%Th	MCM + 2%Th	*p*-Value
AC	0	0.00 ^cd^	0.00 ^cd^	0.00 ^cd^	0.00 ^cd^	0.00 ^cd^	0.00 ^cd^	0.00 ^cd^	0.00 ^cd^	0.00
	1	1.37 ^bA^ ± 0.1	0.06 ^c^ ± 0.2	−1.02 ^cdefg^ ± 0.9	−2.07 ^fghijA^ ± 0.2	−0.82 ^cde^ ± 0.6	−1.5 ^ijA^ ± 1.0	−0.77 ^cde^ ± 0.6	−2.25 ^ghij^ ± 0.8	
	4	4.36 ^aA^ ± 0.2	−1.17 ^defgh^ ± 0.2	−0.68 ^cdefA^ ± 0.5	−0.93 ^efghiA^ ± 0.9	−1.08 ^cdefg^ ± 0.4	−3.22 ^iA^ ± 0.4	−1.48 ^efghi^ ± 0.8	−2.44 ^ij^ ± 0.3	
	7	4.96 ^aA^ ± 0.2	1.48 ^bA^ ± 0.2	−1.49 ^efghiA^ ± 0.2	−2.05 ^fghij^ ± 0.3	−1.58 ^efghi^ ± 0.6	−4.75 ^jA^ ± 0.1	−2.1 ^bcdA^ ± 1.3	−2.34 ^hijA^ ± 0.3	
VC	0	0.00 ^a^	0.00 ^a^	0.00 ^a^	0.00 ^a^	0.00 ^a^	0.00 ^a^	0.00 ^a^	0.00 ^a^	0.00
	1	−0.34 ^abcdB^ ± 0.6	−0.18 ^ab^ ± 0.2	−0.45 ^abcde^ ± 0.2	−0.86 ^abcdefgB^ ± 0.5	−0.65 ^abcdef^ ± 0.9	−0.96 ^abcdefgB^ ± 1.0	−1.46 ^efg^ ± 0.5	−2.07 ^g^ ± 0.1	
	4	−0.43 ^abcdeB^ ± 0.6	−0.51 ^abcdef^ ± 0.8	−1.43 ^defgB^ ± 0.5	−1.02 ^abcdefgB^ ± 0.3	−1.24 ^cdefg^ ± 0.7	−0.94 ^abcdefgB^ ± 0.0	−0.81 ^abcdef^ ± 0.3	−1.65 ^fg^ ± 0.4	
	7	−0.29 ^abcdB^ ± 0.1	−0.25 ^abcB^ ± 0.9	−0.54 ^abcdefB^ ± 0.3	−1.55 ^efg^ ± 0.8	−1.18 ^bcdefg^ ± 0.6	−1.02 ^abcdefgB^ ± 0.8	−0.87 ^abcdefB^ ± 1.0	−1.4 ^cdefgB^ ± 0.2	
AC X VC	0	NS	NS	NS	NS	NS	NS	NS	NS	
	1	<0.0001	0.143	NS	<0.0001	NS	0.005	NS	NS	
	4	<0.0001	0.066	0.026	<0.0001	NS	0.008	NS	NS	
	7	<0.0001	0.002	0.002	NS	NS	<0.0001	0.008	0.008	

a–j Different letters indicate significant differences between days and the treatments (*p* < 0.05). A,B Populations under the same treatments in each column followed by different letters are significantly different (*p* < 0.05).—Initial quantity (N0) of camel meat (CM) in aerobic and vacuum packaging: 4.9 ± 0.3 and 5.2 ± 0.1 log CFU/g; marinated camel meat (MCM) in aerobic and vacuum packaging: 4.7 ± 0.6 and 6.1 ± 0.3 CFU/g.—MCM + 1% Ca: marinated camel meat and 1% carvacrol; MCM + 2% Ca: marinated camel meat and 2% carvacrol; MCM + 1% Ci: marinated camel meat and 1% cinnamaldehyde; MCM + 2% Ci: marinated camel meat and 2% cinnamaldehyde; MCM + 1% Th: marinated camel meat and 1% thymol; MCM + 2% Th: marinated camel meat and 2% thymol. Significantly different at (*p* < 0.05), NS—non-significant.
